# Chimpanzees (*Pan troglodytes*) Indicate Mammalian Abundance Across Broad Spatial Scales

**DOI:** 10.1002/ece3.71000

**Published:** 2025-03-15

**Authors:** Paul K. Kazaba, Lars Kulik, Ghislain B. Beukou Choumbou, Christelle B. Douhin Tiémoko, Funmilayo L. Oni, Serge A. Kamgang, Stefanie Heinicke, Inza Koné, Samedi Jean Pierre Mucyo, Tenekwetche Sop, Christophe Boesch, Colleen Stephens, Anthony Agbor, Samuel Angedakin, Emma Bailey, Mattia Bessone, Charlotte Coupland, Tobias Deschner, Paula Dieguez, Anne‐Céline Granjon, Briana Harder, Josephine Head, Thurston Cleveland Hicks, Sorrel Jones, Parag Kadam, Ammie K. Kalan, Kevin E. Langergraber, Juan Lapuente, Kevin C. Lee, Laura K. Lynn, Nuria Maldonado, Maureen S. McCarthy, Amelia C. Meier, Lucy Jayne Ormsby, Alex Piel, Martha M. Robbins, Lilah Sciaky, Volker Sommer, Fiona A. Stewart, Jane Widness, Roman M. Wittig, Erin G. Wessling, Mimi Arandjelovic, Hjalmar Kühl, Yntze van der Hoek

**Affiliations:** ^1^ Dian Fossey Gorilla Fund, Grauer's Gorilla Research and Conservation Programs Goma Democratic Republic of the Congo; ^2^ Ecology, Restoration Ecology and Landscape (EREP) Research Unit, Département d'Aménagement des Ecosystèmes et Biodiversité, Faculté des Sciences Agronomiques Université de Lubumbashi Lubumbashi Democratic Republic of the Congo; ^3^ Senckenberg Museum for Natural History Görlitz Senckenberg – Member of the Leibniz Association Görlitz Germany; ^4^ World Wide Fund for Nature (WWF), Regional Office for Africa/Cameroon Country Program Office Yaoundé Cameroon; ^5^ Laboratoire de Biodiversité et Ecologie Tropicale Université Jean Lorougnon Guédé Daloa Côte d'Ivoire; ^6^ Department of Wildlife and Ecotourism Management, Faculty of Renewable Natural Resources Ladoke Akintola University of Technology Ogbomoso Nigeria; ^7^ Biodiversité‐Environnement et Développement Durable Garoua Cameroon; ^8^ ERAIFT‐UNESCO Kinshasa Democratic Republic of the Congo; ^9^ Potsdam Institute for Climate Impact Research (PIK), Member of the Leibniz Association Potsdam Germany; ^10^ Université Félix Houphouët‐Boigny Abidjan Côte d'Ivoire; ^11^ Centre Suisse de Recherches Scientifiques en Côte d'Ivoire Abidjan Côte d'Ivoire; ^12^ Dian Fossey Gorilla Fund, Ellen DeGeneres Campus Kinigi Rwanda; ^13^ Re:Wild Austin Texas USA; ^14^ Max Planck Institute for Evolutionary Anthropology Leipzig Germany; ^15^ Department of Environmental Management Makerere University Kampala Uganda; ^16^ Department of Biology, Centre for the Advanced Study of Collective Behaviour University of Konstanz Konstanz Germany; ^17^ Department of Animal Societies Max Planck Institute of Animal Behaviour Konstanz Germany; ^18^ Comparative BioCognition Institute of Cognitive Science, University of Osnabrück Osnabrück Germany; ^19^ German Centre for Integrative Biodiversity Research (iDiv) Leipzig Germany; ^20^ The Biodiversity Consultancy Cambridge UK; ^21^ The Faculty of “Artes Liberales” University of Warsaw Ulica Dobra Warsaw Poland; ^22^ The David Attenborough Building, RSPB Centre for Conservation Science Cambridge UK; ^23^ Warnell School of Forestry and Natural Resources University of Georgia Athens Georgia USA; ^24^ Department of Anthropology University of Victoria Victoria British Columbia Canada; ^25^ School of Human Evolution and Social Change Arizona State University Tempe Arizona USA; ^26^ Institute of Human Origins, Arizona State University Tempe Arizona USA; ^27^ Animal Ecology and Tropical Biology, Biozentrum, (Zoologie III) Würzburg Germany; ^28^ Hawai'i Institute of Marine Biology University of Hawai'i at Mānoa Honolulu Hawaii USA; ^29^ Department of Anthropology University College London London UK; ^30^ Department of Primate Behavior and Evolution Max Planck Institute for Evolutionary Anthropology Leizpig Germany; ^31^ Gashaka Primate Project Serti Taraba Nigeria; ^32^ School of Biological and Environmental Sciences Liverpool John Moores University Liverpool UK; ^33^ Yale University New Haven Connecticut USA; ^34^ Ape Social Mind Lab Institute of Cognitive Science, CNRS UMR5229 Bron France; ^35^ Taï Chimpanzee Project Centre Suisse de Recherche Scientifique en Côte d'Ivoire Abidjan Côte d'Ivoire; ^36^ Cognitive Ethology Laboratory German Primate Center—Leibniz Institute for Primate Research Göttingen Germany; ^37^ International Institute Zittau, Technische Universität Dresden Zittau Germany

**Keywords:** Africa, camera traps, great apes, indicator species, mammals, protected areas

## Abstract

Ongoing ecosystem change and biodiversity decline across the Afrotropics call for tools to monitor the state of biodiversity or ecosystem elements across extensive spatial and temporal scales. We assessed relationships in the co‐occurrence patterns between great apes and other medium to large‐bodied mammals to evaluate whether ape abundance serves as a proxy for mammal diversity across broad spatial scales. We used camera trap footage recorded at 22 research sites, each known to harbor a population of chimpanzees, and some additionally a population of gorillas, across 12 sub‐Saharan African countries. From ~350,000 1‐min camera trap videos recorded between 2010 and 2016, we estimated mammalian community metrics, including species richness, Shannon diversity, and mean animal mass. We then fitted Bayesian Regression Models to assess potential relationships between ape detection rates (as proxy for ape abundance) and these metrics. We included site‐level protection status, human footprint, and precipitation variance as control variables. We found that relationships between detection rates of great apes and other mammal species, as well as animal mass were largely positive. In contrast, relationships between ape detection rate and mammal species richness were less clear and differed according to site protection and human impact context. We found no clear association between ape detection rate and mammal diversity. Our findings suggest that chimpanzees hold potential as indicators of specific elements of mammalian communities, especially population‐level and composition‐related characteristics. Declines in chimpanzee populations may indicate associated declines of sympatric medium to large‐bodied mammal species and highlight the need for improved conservation interventions.Changes in chimpanzee abundance likely precede extirpation of sympatric mammals.

## Graphical abstract

To evaluate wether ape detection rate serves as proxy of mammal community characteristics across broad spatial scales, we used ~350,000 1‐min camera trap videos recorded at 22 sites, each known to harbor a population of chimpanzees, and some also gorillas, across 12 sub‐Saharan African countries. We estimated mammalian community metrics, fitted Bayesian Regression Models, and found that chimpanzees hold potential as indicators of specific mammalian communit traits.

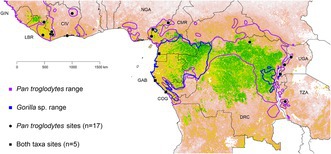

## 1 Introduction

Globally, overexploitation of natural resources, land conversion, climate change, and other anthropogenic factors are contributing to the currently unprecedented high levels of ecosystem degradation and the accompanying rapid declines in biodiversity (Almond et al. 2020; Bergstrom et al. 2021; IPBES 2019; Liu et al. 2007). Complex interactions between components of global change are compounding these problems (Tobias et al. 2021) and are resulting in the emergence of: (i) novel cascading effects, e.g., herbivores may alter their behavior following the extirpation of large carnivores, which subsequently impacts vegetation (Atkins et al. 2019); (ii) negative feedback loops, e.g., a loss of elephants may reduce dispersal of fruiting trees, thereby limiting the capacity of forests to sustain elephant populations (Bush et al. 2020), and (iii) increased risks to ecosystem and human health, e.g., global change may increase the emergence and spread of infectious diseases (Williams et al. 2021). The effects of global changes are also evident in the Afrotropics, where anthropogenically induced loss of biodiversity, deterioration of ecological processes, and collapse of ecosystem functioning reduce the capacity of ecosystems to provide globally important services, such as carbon storage (Dargie et al. 2017; Hubau et al. 2020; Malhi et al. 2013; Midgley and Bond 2015). Worsening problems, efforts to halt or reverse ecosystem collapse, and biodiversity loss are particularly complicated in the Afrotropics, where even endeavors to monitor biodiversity are challenging (see e.g., Anthony et al. 2015; Collen et al. 2008; Nielsen et al. 2009; Siddig 2019). Although it is difficult to measure and monitor the entirety of Afrotropical biodiversity, we may look at surrogates or proxies to gauge the state of aspects of African biodiversity or ecosystems (e.g., community completeness and integrity) across extensive spatial and temporal scales (Hunter et al. 2016; Lindenmayer and Likens 2011).

Nonhuman African great apes (from here on African great apes) are a relatively well‐monitored taxonomic group and may hold promise as indicators of biodiversity across broad spatial scales (see also Stokes et al. 2010). Indeed, African great apes are increasingly impacted by human disturbances and threats, rendering all extant taxa endangered or critically endangered (Fruth et al. 2016; Humle et al. 2016; Kühl et al. 2017; Maisels et al. 2018; Plumptre et al. 2019). In fact, a host of species traits such as high levels of arboreality, dependency on intact and structurally diverse forests, and weak dispersal capacities make apes particularly sensitive to threats from global change and render them possible early sentinels of biodiversity loss (de Almeida‐Rocha et al. 2017; Carvalho et al. 2021). As such, an understanding of the relationships between African apes and other biodiversity, in terms of patterns of co‐occurrence, would potentially allow us to determine how threats and ecosystem change affect apes, and whether these are similar to how other species are affected. There are also additional reasons to consider apes as indicators or management surrogates: First, the charismatic flagship capacity of apes stimulates public and research interest (along with regulation changes), as well as funding (Albert et al. 2018; Marshall et al. 2016), which subsequently generates a wealth of data across large spatial scales (e.g., Heinicke et al. 2021). Second, apes play important roles in ecosystem functioning and stability (e.g., via seed dispersal, Aguado et al. 2022), to the point that the removal of apes may have a cascading effect on forest ecology—as can be the result of the removal of a keystone species (Koffi et al. 2022; Lambert 2011). Third, the ecological requirements of apes render them candidate umbrella species for conservation. Specifically, the protection of relatively vast ape habitats (e.g., Moore et al. 2018; Vieira et al. 2019) can benefit other species with similar habitat requirements and overlapping spatial distributions (Caro 2003).

The abundance of apes in an area may be indicative of the conservation status of other species, partially because apes may play direct causative roles in sustaining biodiversity and ecosystems (Aguado et al. 2022) but mainly because of the correlative effects linked to patterns of co‐occurrence. African apes are predominantly found in relatively intact habitats with low levels of human intervention, habitat degradation, and hunting pressures; areas that are, therefore, also likely to be high in biodiversity (Ordaz‐Németh et al. 2021). Conversely, ape abundances are reduced in areas with high levels of human disturbance (Strindberg et al. 2018); disturbances that are also likely to negatively affect other mammals.

Here, we used data derived from camera trap footage across several Western, Central, and East African great ape range countries to assess the correlative relationships between proxies for ape abundance (using chimpanzees *Pan troglodytes* and gorillas *Gorilla* sp.) and selected aspects of mammal communities. Specifically, we aimed to assess the direction and strength of the relationship between gorilla or chimpanzee detection rate (as a proxy of abundance) and the species richness, the Shannon diversity index (a measure of compositional evenness), detection rate, and animal mass of the mammal community. Data availability drove our selection towards medium‐ to large‐bodied terrestrial mammal (hereafter simplified as “mammal”) communities as response “indicanda” (indicated aspects of biodiversity) of mammals that are likely to share with apes potential responses to anthropogenic threats and are critically important to ecosystem functioning (Rija et al. 2020).

We predicted that the relationships would be positive for both taxa of great apes, as both are more abundant in better‐protected and more intact habitats that have relatively few human disturbances and, therefore, high levels of biodiversity (Strindberg et al. 2018). We also predicted that ape‐mammal relationships are unlikely to be ubiquitous, applicable, or generalizable under all conditions. Specifically, we hypothesized that ape‐mammal relationships would vary spatially under the influence of confounding environmental correlates and variation in local environmental conditions, such as human impact or area protection status (Ekroos et al. 2013). We also considered that there could be interspecific differences in the strength of these relationships: chimpanzees and gorillas differ in their potential as indicator species because of their differences in ecological niches, ecological traits, and responses to threats (Isaac and Cowlishaw 2004; Lehmann et al. 2010; Stanford and Nkurunungi 2003). Although it is important not to oversimplify or overgeneralize ecologies across these species, each of which shows considerable intraspecies variation across their range (e.g., Stanford 2006; van Leeuwen et al. 2020; Wessling et al. 2018), chimpanzees tend to be largely frugivorous (Head et al. 2011) and are, therefore, more dependent on intact habitats with abundant fruit resources (Potts et al. 2009), while the tendency of gorillas to forage on terrestrial or aquatic herbaceous vegetation allows them to persist in more heterogeneous habitats—even if such heterogeneity sometimes stems from human activities (Morgan et al. 2018). In addition, chimpanzees may have a slightly weaker, or more variable, relationship with the indicanda, as they range across both forest and savanna habitats, thus co‐occurring with mammal communities of varying compositions, and may even be found in largely human‐disturbed and fragmented habitats that have chiefly been depleted of other mammals (Bessa et al. 2015; Heinicke et al. 2019; Hicks et al. 2014; Hockings and McLennan 2016; Laudisoit et al. 2021; McCarthy et al. 2017). The Gishwati forest in Rwanda provides a typical example of chimpanzees surviving in a defaunated forest (Sun et al. 2022). Gorillas are, in contrast, more restricted to forests and thus more likely to be limited to larger, more intact tracts of forest with spatially homogeneous mammal communities (i.e., less variation in composition; Strindberg et al. 2018). These interspecific differences, however, in factors such as habitat use and sensitivity to human disturbances are neither universal nor without nuance. For example, chimpanzees in certain regions are more reliant on high‐canopy (and thus undisturbed) forests than gorillas (Morgan et al. 2018; Strindberg et al. 2018). As such, arguments can also be made that the abundance of chimpanzees is a better proxy for the intactness of habitats and mammal communities, and we opted to explore possible interspecific variation in the tested relationships without adhering to a specific prediction.

We also hypothesized that the strength of the relationship between mammal and African ape detection rates would differ across response variables. Specifically, following the typical chronology of defaunation (for a summary see Young et al. 2016), we first predicted that ape detection rates would have a very strong relationship with the total detection rate and animal mass of mammal communities. We may find that the detection rates of apes and other mammals are high in intact areas with little human disturbance and reduced in areas with substantial hunting pressure following a simple linear relationship; an effect exacerbated by other disturbances such as logging (Poulsen et al. 2011). Not all mammals, however, are likely to be affected equally by human pressure, and it is known that large‐bodied species—apes included—are particularly sensitive to disturbances and targeting by hunters (Fritz et al. 2009; Benítez‐López et al. 2019). Thus, ape densities are more likely to mirror the densities of large rather than small mammals at a given site. Such a pattern may also strengthen the relationship between ape detection rates and animal mass.

Next, we predicted that relationships between ape detection rate and other mammal species richness would also be positive. Such positive relationships should become apparent if human pressures affect both declines in ape abundance and the local extirpation of some mammals (see e.g., Whytock et al. 2021). Additionally, such patterns could be linked to spatial patterns in “natural factors,” as may be observed in forest‐savanna transitional landscapes such as the Batéké Plateau in Gabon (Hedwig et al. 2018), where relatively small numbers of great apes and low species richness seem to be partially driven by factors such as plant productivity. Such an explanation would align with the “more individuals hypothesis” that “natural” spatial gradients in factors such as productivity determine both species abundances and richness (as reviewed by Evans et al. 2005). Finally, as apes tend to be abundant in relatively intact landscapes with few human disturbances (Strindberg et al. 2018), landscapes which are also likely to hold relatively intact and diverse mammalian communities (Ahumada et al. 2011), we predicted that sites with high ape detection rate would also have high mammalian diversity.

We considered that the tested relationships could also be influenced by possible confounding and correlative effects. First, as we predicted that ape detection rate and biodiversity richness would be disproportionately and homogeneously high inside protected areas (Gray et al. 2016), we expected that it would be more challenging to detect relationships across sites located inside versus those outside protected areas; we would expect the latter sites to have sampling variance. Second, we hypothesized that comparable responses of great apes and other biodiversity to human disturbances could be one driver of positive ape‐biodiversity relationships and predicted relationships to be stronger in areas with higher levels of human disturbance (see also Ordaz‐Németh et al. 2021). Finally, we determined whether vegetation type would influence the investigated relationships, hypothesizing that ape‐mammal relationships would be less strong in forests than in savanna‐like vegetation, as forests are likely to be more homogeneously diverse in mammals (Turpie and Crowe 1994). We tested this by including inter‐annual precipitation variance (i.e., seasonality), a useful proxy to distinguish between more (savanna‐dominated) and less variable (forest) environmental conditions and associated vegetation (see also Kalan et al. 2020), as covariates in our models.

## 2 Methods

### 2.1 Study Area and Data Collection

**FIGURE 1:**
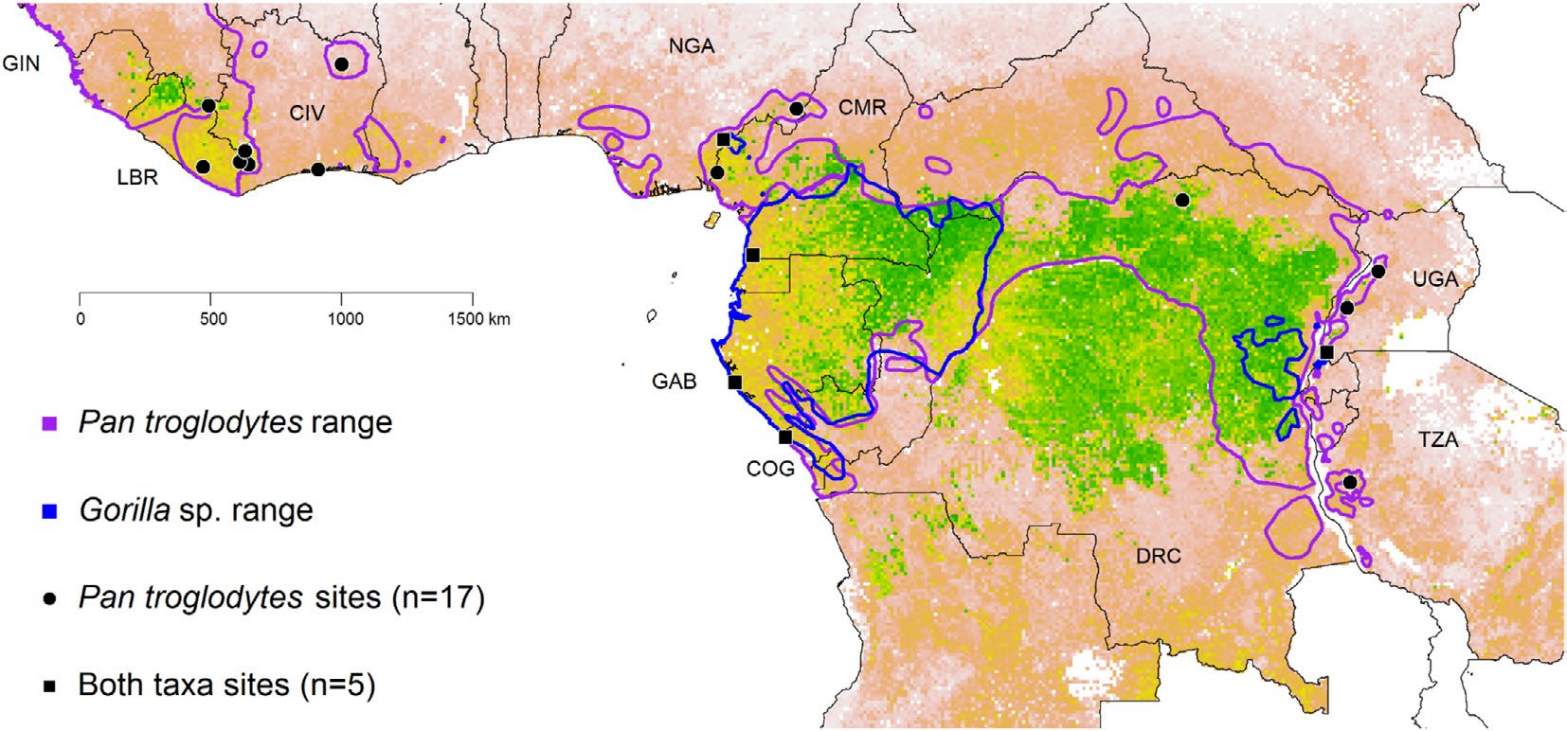
Location of PanAf study sites included in this study (*n* = 22). Colored polygons indicate the approximate ranges of chimpanzee *Pan troglodytes* (Humle et al. 2016) and gorilla [ *Gorilla beringei* (Plumptre et al. 2019) and *Gorilla gorilla* (Maisels et al. 2018)]. The background represents estimated aboveground carbon density derived from Baccini et al. (2012) as a proxy for ecological conditions. Countries included in this study are labeled on the map following standard ISO alpha‐3 codes.

We utilized camera trap data collected between 2010 and 2016 at 22 research sites located in 12 African countries (Figure 1 and Table S1). These data, which were collected between 744 and 10,519 camera trap days at each of the 22 temporary (with unhabituated chimpanzees) and established (with well‐studied chimpanzees) research sites, followed a grid‐based (1 × 1 km^2^) sampling design under the umbrella of the Pan African Programme: the Cultured Chimpanzee (hereafter PanAf; Arandjelovic et al. 2014).

The Pan African Programme aims to understand evolutionary‐ecological drivers that have generated intraspecific diversity in chimpanzees (using data on various populations over their natural range) across Africa. Data were collected within a “data collection zone,” i.e., an area within a 20–100 km^2^ grid with cell size of 1 × 1 km. If no chimpanzee community was known at the site, the field team firstly conducted a recce survey to identify hotspots of chimpanzee activity. Then, once the hotspots identified, they placed the grid with 1 × 1 km cell size and centered it on a hotspot of chimpanzee activity. The camera traps (512 devices in total; Table S1) were mounted at a height ranging between 0.9 and 1.1 m, onto trees with at least 5 cm of diameter at breast height. The maximum recording time was 60 s [see Arandjelovic et al. (2014, 2024) for additional methodological details]. Apes were found at all sites, with *n* = 5 and *n* = 22 sites with gorilla or chimpanzee present, respectively. Sites varied in dominant vegetation type (equatorial forests to forest‐savanna mosaics; Figure 1) and protection status (governmentally protected areas, community‐managed areas, and unprotected areas; Table S1).

### 2.2 Data Processing

We extracted animal sightings from ~350,000 1‐min (camera trap) videos which we had previously annotated to high levels of precision (95.4% of annotation accuracy—obtained by comparing classification of video clips by a professional ecologist to community science annotations; see e.g., McCarthy et al. 2021; Arandjelovic et al. 2024), via the web‐based community science platform Chimp&See (Arandjelovic et al. 2016). We first consolidated species identification and taxonomy (following IUCN 2022). We then excluded all records of non‐mammalian taxa (e.g., birds and reptiles), flying or strictly arboreal mammals, and small mammals with a body mass below ~1 kg because animals of this size are often unidentifiable/hardly captured via camera trap (Glen et al. 2013). This resulted in the retention of 107 species for further analyses. For referential comparisons, we also conducted all data analyses on a narrower subset of species (*n* = 76; Table S2), which excluded predominantly arboreal species with low detection probabilities (Moore et al. 2020).

Considering model covariates, we first categorized research sites by their location within or outside of (government or community) protected areas. Next, we obtained, as a proxy for human disturbances, human footprint estimates for 1994–2005 from an existing spatial layer that integrates information on nine data layers related to human population density, land use and infrastructure, and human access across 1‐km^2^ grid cells: the Human Influence Index (Wildlife Conservation Society—WCS, and Center for International Earth Science Information Network—CIESIN—Columbia University 2005). Specifically, we derived the maximum values for human footprints as they were found within 10‐km radius buffers around the camera traps deployed at a given site. We chose 10 km for these buffers given this is a distance up to which human activities are likely to affect wildlife (see e.g., Torres et al. 2016) and a distance often used in analyses of human activities at the edges of protected areas (e.g., Joppa et al. 2009). Finally, we used inter‐annual precipitation variance (i.e., seasonality) between 1960 and 1990 to distinguish between savanna‐like dominated vegetation—characterized by higher rainfall fluctuations—and relatively more precipitation‐stable equatorial forests (see also Kalan et al. 2020). We obtained estimates for precipitation variance from the WorldClim Precipitation Seasonality (Coefficient of Variation) bioclimatic variable (https://www.worldclim.org/). Low values of precipitation variance are associated with equatorial forest biomes, where there is high rainfall year‐round, and high values (i.e., high seasonal climatic variability) indicate more savanna‐like biomes, many of which are located at higher latitudes.

### 2.3 Data Analyses

We first calculated site‐specific values for predictor, response, and control variables. We utilized the detection rate, i.e., number of daily sightings of a given species as proxies for the local mammalian abundances (hereafter “abundance”) (Palmer et al. 2018). This detection rate‐based method has some limitations when estimating unmarked animals (Burton et al. 2015; Fisher et al. 2020; Hofmeester et al. 2019), but is usable when the objective is to give an indication of relative (not absolute) abundance and guide management efforts (Gilbert et al. 2021), like in this study. The detection rates of gorillas and chimpanzees served as predictor variables and were measured in the same way as for other mammalian species. For response variables, we considered: (i) the sum of the detection of all mammals excluding gorillas and chimpanzees per day; (ii) the (log‐transformed) mean body mass (kg) of all observed medium and large mammal species per day (hereafter “animal mass”); (iii) the observed species richness (count of all species of medium and large mammals observed at least once at a given site); and (iv) the Shannon diversity, estimated using the abundance proxy. We focused on Shannon diversity as a response variable to assess ape‐mammalian diversity relationships because it is relatively sensitive to variation in both richness and evenness (Konopiński 2020). We obtained species‐specific estimates of body mass from mammal traits data sets (Faurby et al. 2020; Smith et al. 2003). We added models with two‐way interaction terms between chimpanzee detection rate and one of three additional predictors (protection, human footprint, precipitation variance). These models could not be run for gorillas because there were too few gorillas (*n* = 5) on some sites, which made these models unstable.

Next, we fitted Bayesian Regression Models to each combination of predictor and response variable using the R (version 4.2.3) package “brms” (Bürkner 2017) for chimpanzees. We controlled for spatial autocorrelation by adding an adjustment term based on the *gp* function in “brms,” a Gaussian process that iterates over the spatial coordinates of each site, and included camera trap days as a control for observation effort. We z‐transformed all predictor variables to improve output interpretability (Schielzeth 2010).

We retained all default settings of the *brm* function and ran models across 2000 iterations and four Markov Chain Monte Carlo (MCMC) chains with a warm‐up of 1000 iterations to derive a total of 8000 posterior distributions (Bürkner 2017). We used wide priors with a normal distribution, a mean of zero, and a standard deviation of 10. After we confirmed the accuracy of the MCMC iterations, all results showed convergence, lacked divergent transitions following warm‐up, and had Rhat values below 1.01 (Gelman et al. 2013, 2014) and determined the credible intervals and mean estimates of marginal posterior distributions.

## 3 Results

**FIGURE 2:**
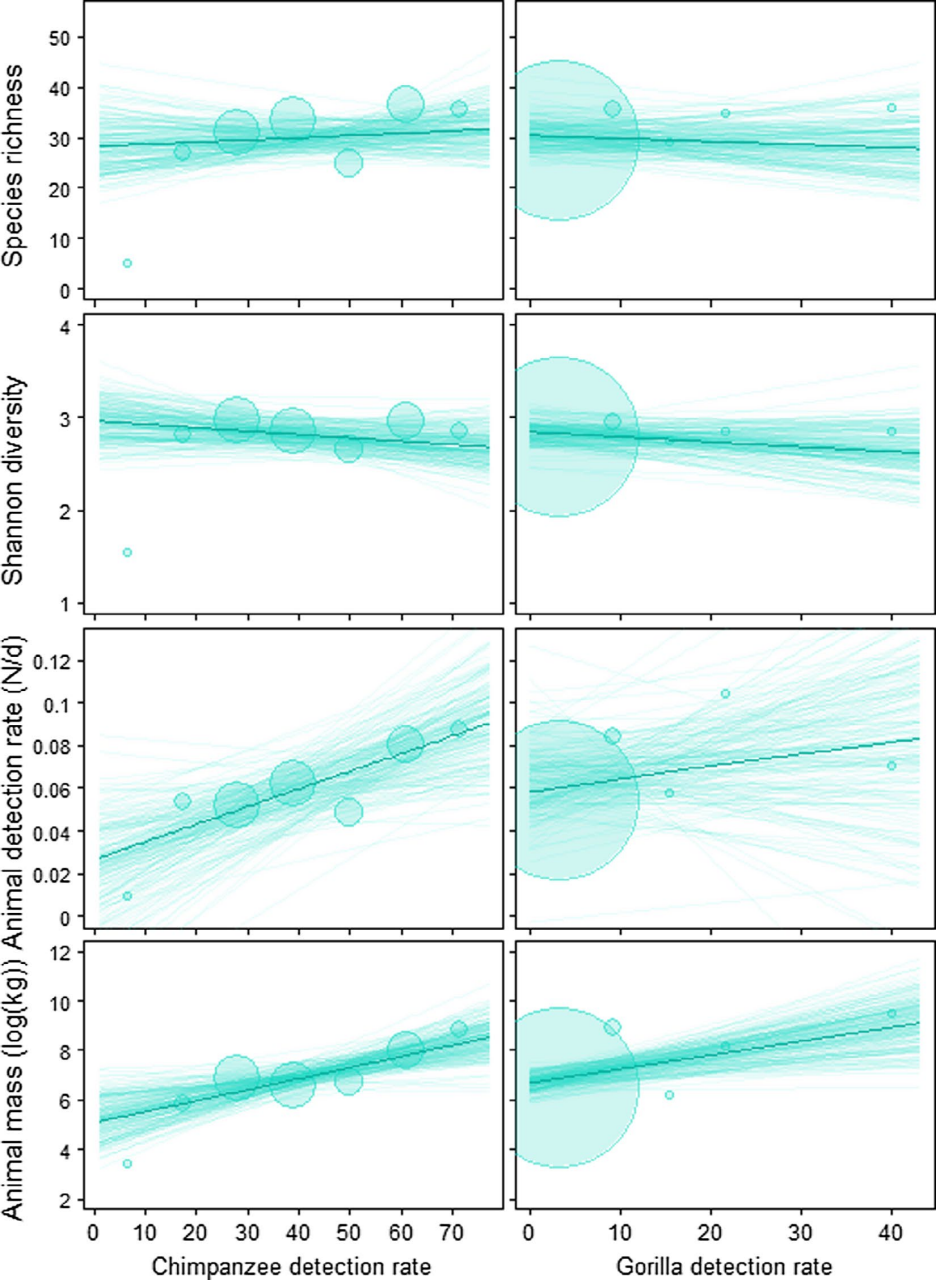
Posterior predictions for the relationship between mammal detection rate, diversity, and species richness, and chimpanzee or gorilla detection rate. Dark continuous lines represent the mean of the posterior distribution, and lighter‐colored lines represent 150 random draws from the posterior. The size of the circles indicates the sample size per value combination.

**FIGURE 3:**
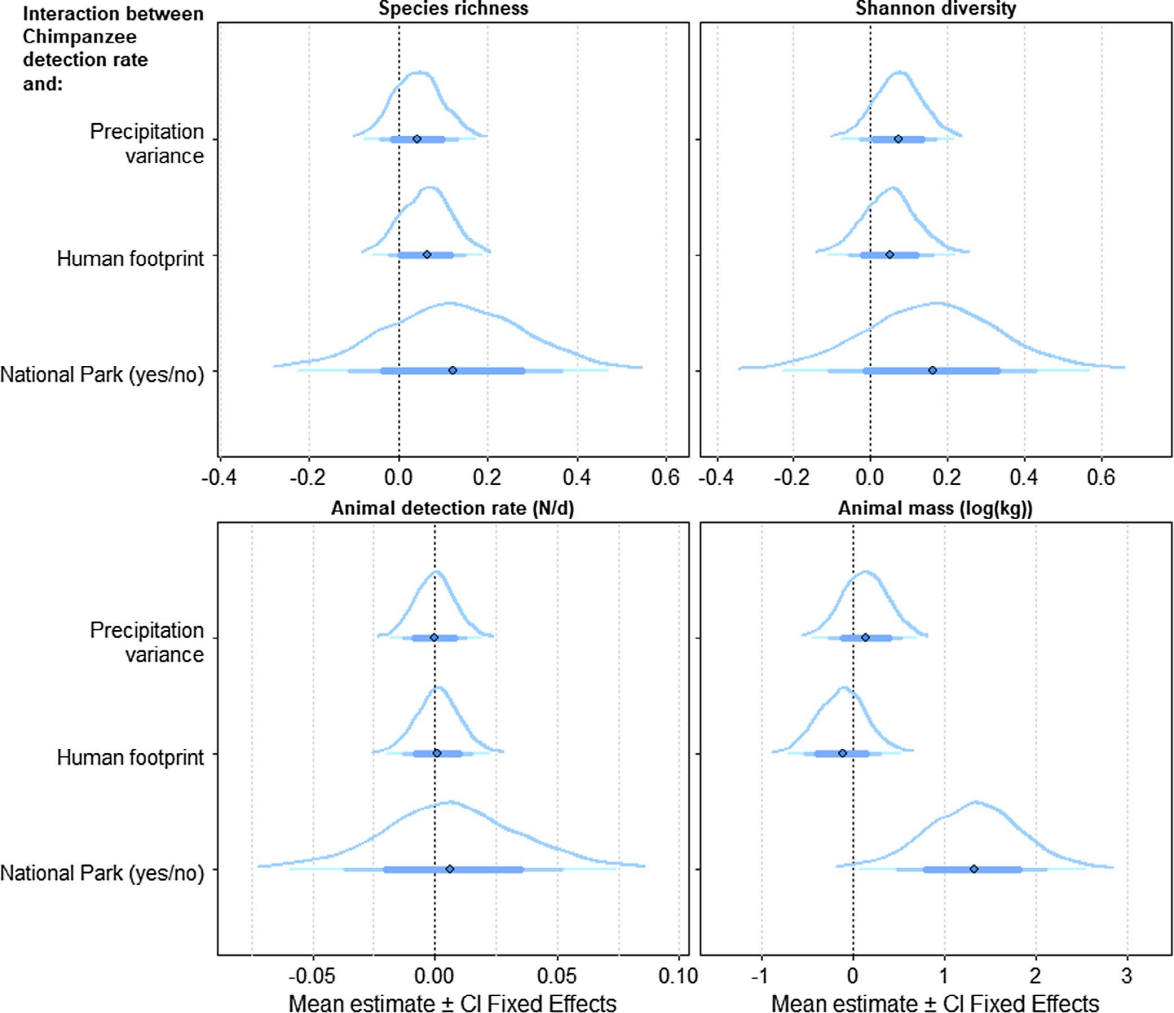
Bayesian Regression Model estimates for posterior distributions of the relationship between chimpanzee detection rates and mammal richness, Shannon diversity, detection rate, and animal mass (kg). Dots represent means of the marginal posterior distributions, the bell curves represent the full posterior distribution, and horizontal lines indicate, in order of line thickness, the 67%, 87%, and 97% credible intervals.

**FIGURE 4:**
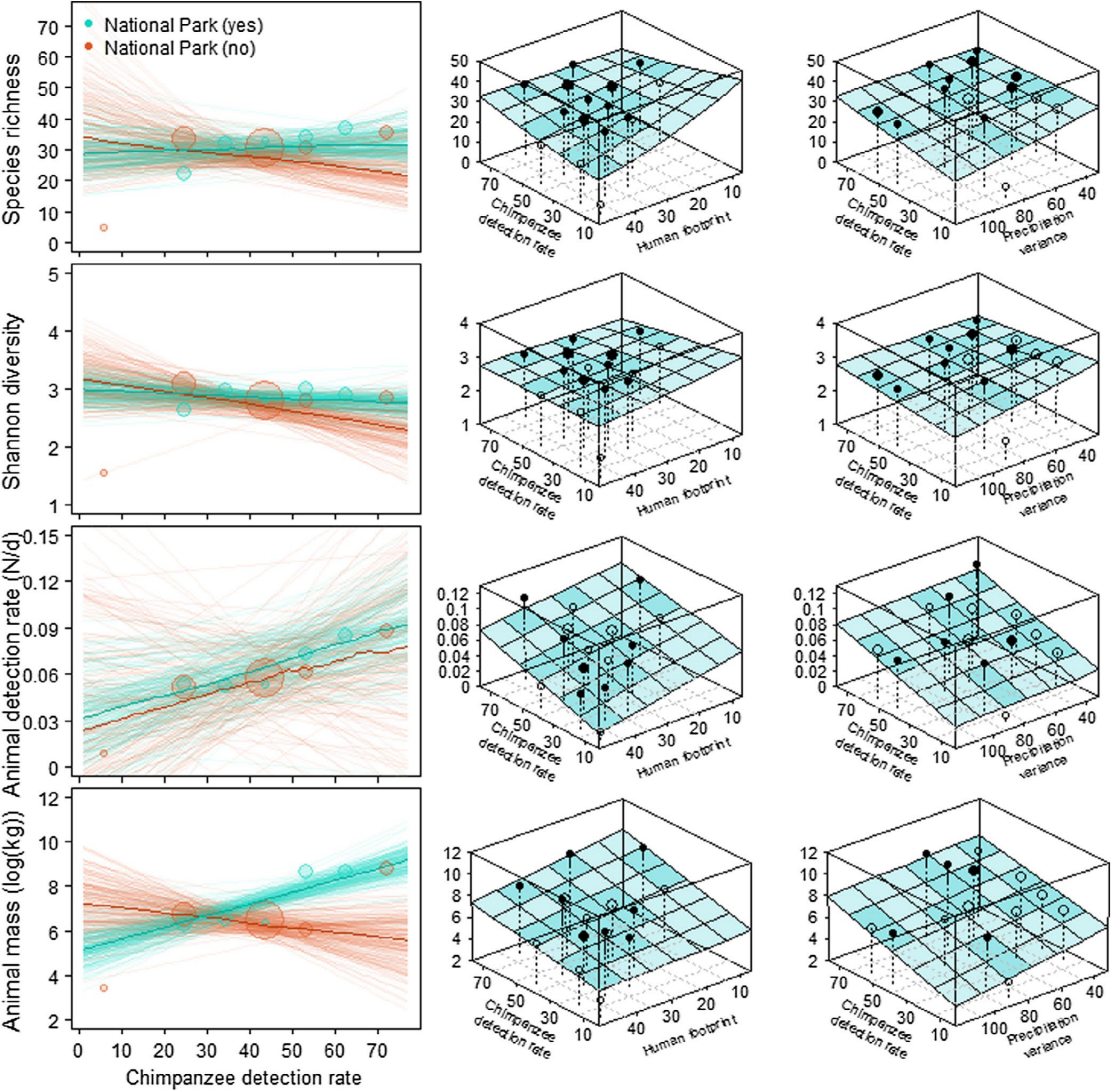
Correlate effects of protection level, human footprint, and precipitation variance on the relationship between chimpanzee ( *Pan troglodytes* ) detection rate and mammal detection rate and diversity. The left‐most, two‐dimensional plots show posterior predictions with dark continuous lines representing mean posterior distributions and lighter‐colored lines representing 150 random draws from the posterior. The size of the circles indicates the sample size per value combination. The three‐dimensional surface plots in the middle and right columns depict continuous marginal posterior distributions, with dots representing the mean, light‐colored cells representing extrapolated estimates, and darker‐colored surface cells for which data was included in the model. Filled points refer to values above fitted model predictions and open points to values below model estimates, with point size corresponding to the available data per cell.

In those places where chimpanzees live, we found that the chimpanzee detection rate was positively related to the overall mammal detection rate (mean marginal posterior distribution ± se = 0.015 ± 0.008; 95% CI = [0.000, 0.031]; Figures 2 and 3 and Table S3); our model predicts an average 23% increase in the mammal detection rate for every additional 10 chimpanzees observed on camera traps. There were no clear signs of either positive or negative relationships between the detection rate of gorillas and other mammals (0.006 ± 0.010; [−0.015, 0.025]). The observed positive relationships between the chimpanzee and mammal detection rates are likely to hold for both protected and unprotected sites, though this relationship has a slightly higher intercept in protected versus unprotected sites (Table S3 and Figure 4). Similarly, we found associations between ape detection rate and animal mass to be positive for both chimpanzees (0.809 ± 0.323; [0.167, 1.437]) and gorillas (0.585 ± 0.258; [0.070, 1.103]). For chimpanzees, these results held in protected but not in unprotected sites (Table S2 and Figure 4). In protected sites with increasing detections rates for chimpanzees we found relatively higher animal mass of other mammals, whereas in unprotected sites the animal mass of other mammals declines slightly with higher detection rates of chimpanzees. 

Interaction models that included human footprint or precipitation variance terms suggest that the observed relationships for detection rates and animal mass were negatively influenced by human impact for sites with larger chimpanzee detection rate and are more pronounced in sites with high (savanna‐like) vs (equatorial forests) levels of precipitation variance (Table S3 and Figure 4).

We found no clear relationship between mammal richness and the detection rate of gorillas (−0.022 ± 0.048; [−0.116, 0.067]) or chimpanzees (0.026 ± 0.060; [−0.092, 0.140]) (Figures 2 and 3 and Table S3), likely due to the impact of interacting effects. For example, protection status interacts with the chimpanzee detection rate–richness relationship to the extent that a positive relationship holds for protected but not unprotected sites (0.123 ± 0.158; [−0.195, 0.427]; Figure 4). Similarly, themammal detection rate was positively associated with an increase in the chimpanzee detection rate in areas with high human impacts, where the detection rates for both chimpanzees and mammals were already lower compared to areas with low human footprints (0.001 ± 0.009; [−0.017, 0.020]).

Finally, relationships between ape detection rate and Shannon diversity seemed marginally negative for both chimpanzees (−0.067 ± 0.078; [−0.220, 0.088]) and gorillas (−0.055 ± 0.064; [−0.181, 0.070]). These relationships seemed to largely hold regardless of possible interacting effects (Table S3 and Figure 4). For instance, when accounting for site protection status, there was a negative relationship between chimpanzee detection rate and Shannon diversity in unprotected areas, but only a very weak one in protected areas. This effect seems marginally reversed in savanna‐like (high precipitation variance) areas, but holds in forest‐dominated (low variance) regions (0.072 ± 0.065; [−0.061, 0.202]).

To ensure that our results were not sensitive to subjective cut‐off points in species inclusion criteria, we ran all models again excluding predominantly arboreal species (remaining *n* = 85). As these models gave comparable results, we decided to continue our additional analyses and discussion based on the models that considered the full (*n* = 107) mammalian community (Figure S1 and Table S3).

## 4 Discussion

As predicted, overall, we found support for a positive relationship between African great ape detection rate and mammal detection rate as well as animal mass. However, in contrast with our predictions, we found no consistent positive relationships between ape detection rate and diversity‐related indices. Positive relationships between ape detection rate and richness were only evident for chimpanzees within protected areas, while relationships between ape detection rate and Shannon diversity were weak and largely negative. In other words, the frequency distribution of mammals became more uneven within mammalian assemblages with increasing ape abundance. Also contrary to our predictions, we found little evidence that chimpanzee‐biodiversity relationships vary across landscapes with different degrees of human disturbance, although there were weak indications of a more positive chimpanzee‐richness relationship in areas with high versus low levels of human disturbance. Finally, the tested relationships varied little across the savanna‐forest (i.e., precipitation variance) gradient, although our models hint at a possible negative chimpanzee‐diversity relationship across savanna‐like sites not dominated by forests.

Positive relationships between great ape detection rate and animal detection rate are in line with the idea that spatial patterns in natural environmental variability [e.g., those linked to food availability (East 1984)] shape site‐specific carrying capacities and population sizes of both great apes (chimpanzees and gorillas) and other mammals. In addition, this relationship may be strengthened if the effects of human‐induced defaunation (e.g., via bushmeat hunting) reduce the populations of both apes and other mammals at comparable rates, with low ape and mammalian densities at sites with high hunting pressure and high densities at sites with low hunting pressure. Evidence of the effects of defaunation on ape–mammal relationships is also apparent when we consider that sites with low ape detection rates have a low animal mass; an observation that is potentially indicative of the disproportionate removal of large‐bodied mammals following human disturbances (Benítez‐López et al. 2019).

Although areas with high ape detection rate seem indicative of areas with high mammal densities and relatively intact communities, there are multiple reasons why areas with high ape detection rate are not always more diverse. First, apes are protected culturally (Etiendem et al. 2011) or are often a focus of law enforcement attention to greater depths than other sympatric mammals (Pailler et al. 2009). Therefore, they can be found persisting in landscapes where other sympatric wildlife have been extirpated or greatly reduced (Boesch et al. 2017; Heinicke et al. 2019). Second, we may reason that positive relationships between detection rate and richness should be apparent even in the absence of a gradient of human disturbances, especially if we consider the “more individuals hypothesis,” which states that factors such as plant productivity drive abundance and subsequently richness (Storch et al. 2018). However, there is mixed support for this hypothesis (see e.g., Storch et al. 2018) and links between productivity, abundance, and richness may only become apparent under specific conditions and after accounting for natural population variability (Vagle and McCain 2020). Third, although correlations in the way site‐specific abundances, species richness, and diversity change spatially or temporally tend to be positive, they are difficult to generalize (Blowes et al. 2022). Moreover, we only had data available for sites where apes were (still) present and where mammalian richness and diversity were also generally high. This lack of data variance may have further reduced our capacity to detect relationships, as substantial reductions of richness may only be apparent in areas from which apes have already been extirpated. Some tentative evidence for the latter possibility comes from sites not included in our dataset, such as localities in Cameroon where a largely hunting‐driven extirpation of apes was accompanied by a similar loss of other large mammals as well as an overall decline in biodiversity and a reduction of wildlife biomass (Maisels et al. 2001).

The relationships between ape detection rate and Shannon diversity are also difficult to generalize because they show different patterns in savanna and forest‐dominated ecosystems (i.e. sites with respectively high and low precipitation variability). They also vary across sites with low and high human disturbances, as well as between areas with and without formal means of protection. Moreover, intact habitats with high densities of apes are also likely to harbor other large‐bodied mammals; these species generally occur at lower densities than small‐bodied mammals (Fa and Purvis 1997). As such, we might find cases where a high ape density corresponds to a high species richness, but a relatively low evenness of detection rates across species, i.e., a relatively low Shannon diversity, obscuring ape detection rate–Shannon diversity relationships. Indeed, some sites included in our dataset had relatively high species richness but only medium‐high Shannon diversity. This was true, for example, for the Issa Valley in Tanzania, a species‐rich site that has several very scarce species (e.g., wild dogs and lions; Piel et al. 2018). Finally, we acknowledge that spatial patterns of ape abundance and biodiversity may not correlate at the scales at which we tested relationships, because ape abundances are largely determined by local processes (e.g., those related to habitat quality and human disturbance; Strindberg et al. 2018; Carvalho et al. 2022), while the distribution of biodiversity is governed by broader processes of speciation and extinction (see e.g., Faurby and Svenning 2015).

Notably, relationships between chimpanzee detection rates and the larger mammalian communities in which they occur differed between protected and unprotected sites. For example, across protected areas, sites with high chimpanzee detection rates have higher species richness than sites with low chimpanzee detection rates in protected areas, whereas this relationship is likely to be negative in unprotected areas. Similarly, our interaction models suggest that chimpanzee‐mammal richness relationships are more likely to be positive in areas with high rather than low human disturbance. These differences may stem partially from a decoupling of site‐specific temporal changes in abundance versus richness or diversity (Blowes et al. 2022), a hypothesis that can potentially be tested in the future when long‐term data become increasingly available and synthesized across study sites (see e.g., Heinicke et al. 2021). Additionally, these observed interactions may stem from the inclusion of specific sites in our data. For example, many of the unprotected sites included in our studies are smaller and largely isolated chimpanzee habitats in West Africa (Table S1). Although chimpanzees are highly threatened across this region (Kühl et al. 2017), certain populations may persist rather well under specific conditions, specifically in unprotected areas (e.g., where there are hunting taboos for this species; Boesch et al. 2017; Heinicke et al. 2019). As a result, we may find that some areas, such as the Bakoun Classified Forest (now part of the Moyen‐Bafing National Park) in Guinea, have a relatively high chimpanzee abundance while the richness and diversity of other mammals have been reduced by hunting. A relatively high awareness of the protected status of chimpanzees may also limit the hunting of this species (Pailler et al. 2009).

We remain cautious in interpreting any differences between gorillas and chimpanzees in their patterns of co‐occurrence with other mammals, given that we only had gorilla data from a limited number of sites (*N* = 5). Yet overall, we found chimpanzee detection rate served as a more pragmatic proxy for the larger mammalian community, specifically because chimpanzees occur under a wide range of environmental conditions (e.g., Bessa et al. 2015; Heinicke et al. 2019; Hockings and McLennan 2016; Humle et al. 2016; Laudisoit et al. 2021; McCarthy et al. 2017; Wessling et al. 2020), and thus provided us with a sufficient degree of variance in mammalian richness, diversity, abundance, and animal mass across sites. Gorilla occurrence, in contrast, is mostly limited to tropical forests where both mammalian abundance and diversity are high across all sites (Table S1; see also e.g., Gorczynski et al. 2021), thus reducing our capacity to detect any clear relationships.

## 5 Conclusion

Estimates of African great ape abundance have the potential to serve as a surrogate for the management of other large mammal species, as trends in ape populations may reflect trends in the abundance of these other mammals. Our study does not address whether patterns of co‐occurrence between great apes and other terrestrial mammals are shaped by causal or correlative effects, and the tested relationships were likely influenced by sample size limitations, sample location choice, and the confounding effects of a number of other factors (e.g., hunting taboos and the spread of Ebola; Strindberg et al. 2018). Yet, although further investigation into these relationships is warranted, our results allow us to already speak to the potential of great apes—chimpanzees in particular—as proxy indicators for certain metrics of mammalian communities. As declines in wildlife abundance precede extirpations and extinctions (Ceballos et al. 2017), observed declines in ape detection rates may provide an early signal of defaunation and help stir the international conservation community into urgent action.

For further studies or other areas, we recommend identifying similar potential proxy species, great apes or other large mammals, which could serve as indicators of ecosystem health and early warning signals of biodiversity loss. These studies should consider species assemblages, activity patterns and protection status, region‐specific ecological contexts, and conservation challenges (e.g., using data from relatively intact forests to compare as control) to effectively mirror environmental conditions.

## Author Contributions

**Paul K. Kazaba:** conceptualization (equal), formal analysis (equal), methodology (equal), software (equal), validation (equal), writing – original draft (equal), writing – review and editing (lead). **Lars Kulik:** conceptualization (equal), data curation (equal), formal analysis (lead), investigation (equal), methodology (equal), validation (equal), visualization (lead). **Ghislain B. Beukou Choumbou:** methodology (equal), validation (equal), writing – original draft (equal), writing – review and editing (equal). **Christelle B. Douhin Tiémoko:** methodology (equal), validation (equal), writing – original draft (equal), writing – review and editing (equal). **Funmilayo L. Oni:** methodology (equal), validation (equal), writing – original draft (equal), writing – review and editing (equal). **Serge A. Kamgang:** methodology (equal), validation (equal), writing – original draft (equal), writing – review and editing (equal). **Stefanie Heinicke:** conceptualization (equal), formal analysis (equal), supervision (equal), writing – review and editing (equal). **Inza Koné:** conceptualization (equal), supervision (equal), writing – review and editing (equal). **Samedi Jean Pierre Mucyo:** conceptualization (equal), supervision (equal), writing – review and editing (equal). **Tenekwetche Sop:** conceptualization (equal), supervision (equal), writing – review and editing (equal). **Christophe Boesch:** data curation (equal). **Colleen Stephens:** data curation (equal), validation (equal), writing – review and editing (equal). **Anthony Agbor:** data curation (equal), validation (equal), writing – review and editing (equal). **Samuel Angedakin:** data curation (equal), validation (equal), writing – review and editing (equal). **Emma Bailey:** data curation (equal), validation (equal), writing – review and editing (equal). **Mattia Bessone:** data curation (equal), validation (equal), writing – review and editing (equal). **Charlotte Coupland:** data curation (equal), validation (equal), writing – review and editing (equal). **Tobias Deschner:** data curation (equal), validation (equal), writing – review and editing (equal). **Paula Dieguez:** data curation (equal), validation (equal), writing – review and editing (equal). **Anne‐Céline Granjon:** data curation (equal), validation (equal), writing – review and editing (equal). **Briana Harder:** data curation (equal), validation (equal), writing – review and editing (equal). **Josephine Head:** data curation (equal), validation (equal), writing – review and editing (equal). **Thurston Cleveland Hicks:** data curation (equal), validation (equal), writing – review and editing (equal). **Sorrel Jones:** data curation (equal), validation (equal), writing – review and editing (equal). **Parag Kadam:** data curation (equal), validation (equal), writing – review and editing (equal). **Ammie K. Kalan:** data curation (equal), validation (equal), writing – review and editing (equal). **Kevin E. Langergraber:** data curation (equal), validation (equal), writing – review and editing (equal). **Juan Lapuente:** data curation (equal), validation (equal), writing – review and editing (equal). **Kevin C. Lee:** data curation (equal), validation (equal), writing – review and editing (equal). **Laura K. Lynn:** data curation (equal), validation (equal), writing – review and editing (equal). **Nuria Maldonado:** data curation (equal), validation (equal), writing – review and editing (equal). **Maureen S. McCarthy:** data curation (equal), validation (equal), writing – review and editing (equal). **Amelia C. Meier:** data curation (equal), validation (equal), writing – review and editing (equal). **Lucy Jayne Ormsby:** data curation (equal), validation (equal), writing – review and editing (equal). **Alex Piel:** data curation (equal), validation (equal), writing – review and editing (equal). **Martha M. Robbins:** data curation (equal), validation (equal), writing – review and editing (equal). **Lilah Sciaky:** data curation (equal), validation (equal), writing – review and editing (equal). **Volker Sommer:** data curation (equal), validation (equal), writing – review and editing (equal). **Fiona A. Stewart:** data curation (equal), validation (equal), writing – review and editing (equal). **Jane Widness:** data curation (equal), validation (equal), writing – review and editing (equal). **Roman M. Wittig:** data curation (equal), validation (equal), writing – review and editing (equal). **Erin G. Wessling:** data curation (equal), validation (equal), writing – review and editing (equal). **Mimi Arandjelovic:** data curation (lead), methodology (equal), validation (equal), writing – review and editing (equal). **Hjalmar Kühl:** conceptualization (equal), data curation (equal), formal analysis (equal), funding acquisition (lead), investigation (equal), methodology (equal), software (equal), supervision (equal), validation (equal), visualization (equal), writing – original draft (equal). **Yntze van der Hoek:** conceptualization (lead), data curation (equal), formal analysis (equal), investigation (equal), methodology (equal), software (equal), supervision (lead), validation (equal), visualization (equal), writing – original draft (equal), writing – review and editing (equal).

## Conflicts of Interest

The authors declare no conflicts of interest.

## Supporting information


**Table S1.** General information on selected PanAf study sites (*n* = 22).
**Table S2.** List of mammal species (*n* = 76) included in the subset analysis performed for referential comparisons (predominantly arboreal mammals were excluded; see last column of the table).
**Table S3.** Results of Bayesian Regression Models based on weak priors testing the probability of relationships between chimpanzee and/or gorilla detection rate and various metrics of mammalian communities.

## Data Availability

Data and model scripts are available in the Table S1–S3 of this paper or otherwise publicly available.
