# Supplementary figures and images for: Chimpanzees (Pan troglodytes) Indicate Mammalian Abundance Across Broad Spatial Scales
Source: Ecol Evol. 2025 Mar 15;15(3):e71000. doi: 10.1002/ece3.71000 (PMC11909632; doi:10.1002/ece3.71000)

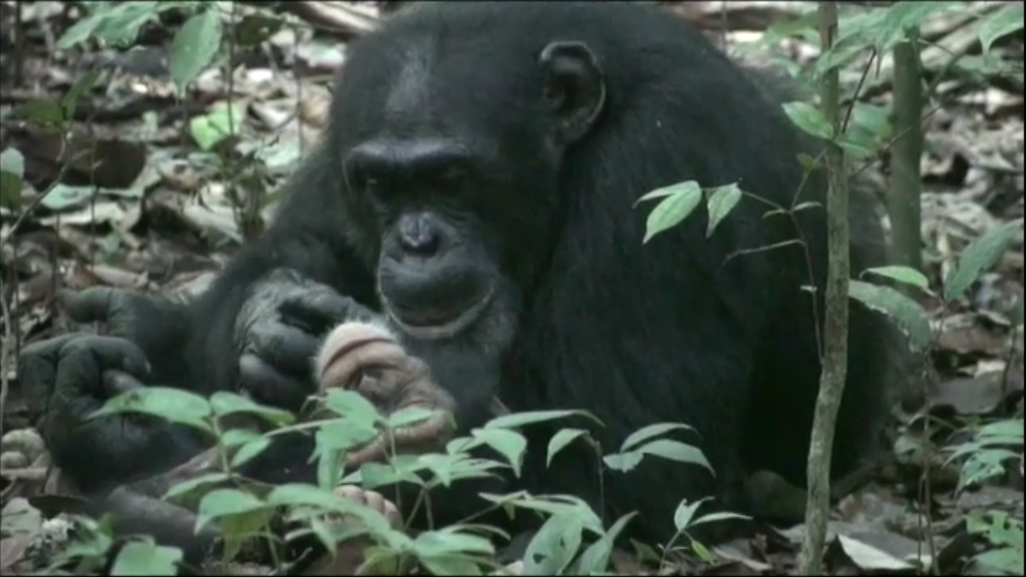

Supplement: Supplementary file 1 — Table S1. General information on selected PanAf study sites (n = 22). Table S2. List of mammal species (n = 76) included in the subset analysis performed for referential comparisons (predominantly arboreal mammals were excluded; see last column of the table). Table S3. Results of Bayesian Regression Models based on weak priors testing the probability of relationships between chimpanzee and/or gorilla detection rate and various metrics of mammalian communities. [file ECE3-15-e71000-s001.zip › ece371000-sup-0001-FigureS1.tif]
